# The osteogenic response of mesenchymal stromal cells to strontium‐substituted bioactive glasses

**DOI:** 10.1002/term.2003

**Published:** 2015-03-11

**Authors:** Martin E. Santocildes‐Romero, Aileen Crawford, Paul V. Hatton, Rebecca L. Goodchild, Ian M. Reaney, Cheryl A. Miller

**Affiliations:** ^1^ School of Clinical DentistryUniversity of SheffieldUK; ^2^ Department of Materials Science and EngineeringUniversity of SheffieldUK

**Keywords:** bioactive glass, strontium, osteogenic differentiation, mesenchymal stromal cells, real‐time PCR

## Abstract

Bioactive glasses are known to stimulate bone healing, and the incorporation of strontium has the potential to increase their potency. In this study, calcium oxide in the 45S5 bioactive glass composition was partially (50%, Sr50) or fully (100%, Sr100) substituted with strontium oxide on a molar basis. The effects of the substitution on bioactive glass properties were studied, including density, solubility, and *in vitro* cytotoxicity. Stimulation of osteogenic differentiation was investigated using mesenchymal stromal cells obtained from rat bone marrow. Strontium substitution resulted in altered physical properties including increased solubility. Statistically significant reductions in cell viability were observed with the addition of bioactive glass powders to culture medium. Specifically, addition of ≥ 13.3 mg/ml of 45S5 bioactive glass or Sr50, or ≥ 6.7 mg/ml of Sr100, resulted in significant inhibition. Real‐time PCR analyses detected the upregulation of genes associated with osteoblastic differentiation in the presence of all bioactive glass compositions. Some genes, including *Alpl* and *Bglap*, were further stimulated in the presence of Sr50 and Sr100. It was concluded that strontium‐substituted bioactive glasses promoted osteogenesis in a differentiating bone cell culture model and, therefore, have considerable potential for use as improved bioactive glasses for bone tissue regeneration. © 2015 The Authors. Journal of *Tissue Engineering and Regenerative Medicine* Published by John Wiley & Sons Ltd.

## 1 Introduction

Since their development by Larry Hench in the 1960s (Hench, 2006), bioactive glasses have been used extensively in the treatment of bone tissue defects, due to their ability to stimulate healing via dissolution, followed by the formation of a surface layer of hydroxycarbonate apatite. Once the surface modification process has occurred, the hydroxycarbonate apatite layer interacts with collagen fibrils in the damaged tissues, creating a bond. The biological interactions leading to this outcome are thought to include the adsorption of proteins and the attachment of cells, followed by their differentiation and production of new extracellular bone matrix (Hench, 1998; Jones, 2013). Various dissolution products released by bioactive glasses (e.g. soluble silica, Ca^2+^) may induce osteogenesis *in vitro*. For example, 45S5 bioactive glass dissolution products can influence the osteoblast cell cycle, resulting in the potential selection of osteoblast subpopulations through the apoptotic death of cells unable to adapt to the microenvironment created by the glass (Xynos *et al*., 2000a). Enhancing effects on human osteoblasts include the *in vitro* promotion of cellular proliferation, a shortened growth cell cycle, and the increased expression of genes associated with metabolism and bone homeostasis (Sun *et al*., 2007; Xynos *et al*., 2000b, 2001). Bioactive glass dissolution products were also observed to stimulate the proliferation of rat bone marrow cells and the production of growth factors and extracellular matrix (Bosetti and Cannas, 2005). Both surface‐ and solution‐mediated events are reported to play a significant role in the osteogenic effect of bioactive glasses (Radin *et al*., 2005).

These studies contrasted with previous reports that bioactive glasses were simply cytotoxic *in vitro*, due to the release of sodium ions that increased the alkalinity of the environment (Wallace *et al*., 1999). Interestingly, Reilly *et al*. (2007) observed that human bone marrow cells responded differently to bioactive glass dissolution products than rat‐derived cells, exhibiting significantly less alkaline phosphatase activity and not responding consistently to the growth factor BMP‐2 under the same experimental conditions. However, Tsigkou *et al*. (2009) reported that bioactive glass conditioned medium was indeed able to promote differentiation of human fetal osteoprogenitor cells into mature osteoblasts, resulting in the production of bone nodules *in vitro*. Additionally, varying degrees of response may also be observed, depending on the type of stem cell. Although human adipose‐derived cells may exhibit greater osteogenic potential than bone marrow‐derived cells (Rath *et al*., 2013), in some cases the presence of the glass did not have an apparent effect on the differentiation of both cell types toward an osteogenic lineage (Tsigkou *et al*., 2014). To summarize, despite extensive study, the degree to which bioactive glasses are cytotoxic *in vitro* vs their ability to stimulate osteogenic differentiation is a question that remains unresolved. Hoppe *et al*. (2011) have reviewed the general role of bioactive glass dissolution products in relation to osteogenesis and angiogenesis; while this review did not address the issue of cytotoxicity, it identified a need for more systematic studies of bioactive glasses.

Strontium has attracted attention due to its reportedly beneficial effects on bone healing. For example, strontium ranelate has proved to be an effective treatment against osteoporosis in postmenopausal women, acting via a mechanism combining the inhibition of bone resorption with the stimulation of new bone tissue formation. Evidence shows that strontium is able to inhibit osteoclast differentiation and activity, while simultaneously inducing osteoclast apoptosis, osteoblast differentiation and the mineralization of new bone matrix (Bonnelye *et al*., 2008; Hurtel‐Lemaire *et al*., 2009; Marie, 2005, 2007; Marie *et al*., 2001). The molecular mechanisms through which this effect is most likely achieved are various and have been reviewed elsewhere by Saidak and Marie (2012). Given the potency of strontium, it was recently suggested that bioactive glasses in which calcium oxide is substituted with strontium oxide may be formulated in order to enhance bone tissue regeneration (Lao *et al*., 2008). A few studies have reported the enhancing effects of strontium‐substituted bioactive glasses on osteogenesis *in vitro* using different cell sources, demonstrating their potential for bone tissue regeneration. Gentleman *et al*. (2010) used the human osteosarcoma cell line Saos‐2 and osteoclasts derived from a mouse monocyte cell line, and Isaac *et al*. (2011) used fetal mouse calvaria osteoblastic cells. Stromal cells were used by Wu *et al*. (2011) in the study of mesoporous bioactive glass, by Strobel *et al*. (2013) in the study of bioactive glass nanoparticles fabricated by flame spray synthesis, and by Zhu *et al*. (2013) in the study of strontium‐substituted borate bioactive glass compositions. Animal studies have also reported the regenerative potential of these glass compositions in various locations of the skeleton *in vivo*, including the tibia (Gorustovich *et al*., 2010), the femur (Jebahi *et al*., 2013; Wei *et al*., 2014) and the periodontium (Zhang *et al*., 2014). Gorustovich *et al*. (2010) reported good bonding of the bioactive glasses to bone tissue, with no differences observed between the unmodified and the strontium‐substituted compositions with regard to their ability to osseo‐integrate. This may have been due to the strontium substitution being done on a weight basis, rather than on a molar basis, which may result in reduced bioactivity. Although Jebahi *et al*. (2013) also performed the substitution on a weight basis, they reported enhanced bone formation and osteoblast recruitment compared with the unmodified glass in osteopenic animals. More recently, Zhang *et al*. (2014) and Wei *et al*. (2014) observed significant regeneration of damaged bone tissue and decreased numbers of multinucleated osteoclasts in osteopenic animals treated with strontium‐releasing mesoporous bioactive glass.

To summarize, a small but growing body of work suggests that strontium‐substituted bioactive glasses may form the basis for the development of improved bone graft substitutes. Therefore, the aim of this study was to investigate the effect of particulate strontium‐substituted bioactive glasses on the metabolic activity and osteogenic differentiation of mesenchymal stromal cells isolated from rat bone marrow, in order to evaluate their potential use in composite materials for bone regeneration applications. Bioactive glasses based on the 45S5 composition were fabricated in which calcium oxide was substituted by strontium oxide on a molar basis. These were characterized using a range of techniques in order to determine how the substitution affected various physical properties. Finally, the *in vitro* effect of the bioactive glasses on the metabolic activity and osteogenic potential of mesenchymal stromal cells was investigated in order to provide a better understanding of their regenerative capacity.

## 2 Materials and methods

### 2.1 Bioactive glass production and processing

Three bioactive glass compositions, in which calcium oxide was substituted by strontium oxide in molar proportions of 0%, 50% and 100% (Table 1), were produced by a melt–quench route, based on 45S5 bioactive glass (Hench, 2006) and compositions published by O'Donnell *et al*. (2010). Analytical grade SiO_2_, CaCO_3_, Na_3_PO_4_ (Fisher Scientific, UK) and SrCO_3_ (Sigma Aldrich, UK) were mixed and melted in platinum crucibles at 1350°C for 180 min, including 120 min of homogenization using a platinum paddle rotating at 60 rpm. Glass blocks were then produced by pouring the melt into graphite moulds, followed by annealing at temperatures in the range 450–500°C, depending on the proportion of strontium substitution and on the glass transition temperatures reported by O'Donnell *et al*. (2010). Bioactive glass powders (<45 µm particle size) were produced by milling and sieving glass frits obtained by rapidly quenching the melt in distilled water.

**Table 1:** Composition of Sr0, Sr50 and Sr100 bioactive glasses, presented in weight percentage (wt%) and molar percentage (mol%)

	Bioactive glasses		
Oxide	Sr0	Sr50	Sr100
*wt%*			
SiO_2_	45.00	40.77	37.26
Na_2_O	24.50	22.19	20.29
CaO	24.50	11.10	0.00
SrO	0.00	20.51	37.49
P_2_O_5_	6.00	5.44	4.97
*mol%*			
SiO_2_	46.13	46.13	46.13
Na_2_O	24.35	24.35	24.35
CaO	26.91	13.46	0.00
SrO	0.00	13.46	26.91
P_2_O_5_	2.60	2.60	2.60

### 2.2 Particle size analysis and scanning electron microscopy (SEM) imaging

Particle size analyses of the bioactive glass powders were performed using static light scattering on a Beckman LS‐130 Coulter device. Each sample was loaded into the system using water as suspension fluid, without deflocculant, and with the ultrasonic mode activated in order to facilitate particle dispersion. Individual measurements were taken in triplicate over a period of 90 s and the acquired data was analysed using Coulter LS software (Beckman Coulter, USA). The morphology of the glass particles was studied using SEM. All samples were sputter‐coated with gold and examined on a Jeol JSM6400 SEM operated at 20 kV.

### 2.3 X‐ray diffraction (XRD) analysis

XRD analyses of bioactive glass powders were performed to confirm the amorphous nature of the materials. All samples were loaded on aluminium sample trays and analysed in a Philips PW1825/00 diffractometer, using Cu radiation, with angles in the range 10–70° 2*θ*, step sizes of 0.02° 2*θ* and scanning speeds of 2°/min. The XRD spectra were processed using WinX^Pow^ software (STOE & CIE GmbH, Germany) and Traces software (GBC Scientific Equipment, Australia).

### 2.4 Differential thermal analysis

Differential thermal analysis (DTA) of the bioactive glass powders was performed to determine their characteristic temperatures. All samples were loaded into platinum crucibles and heated from room temperature to 1200°C at a rate of 10°C/min in a Perkin–Elmer Diamond DTA/TG. The DTA patterns were processed using Pyris software (Perkin‐Elmer, USA) and MjoGraph software (Ochiai Laboratory, Yokohama National University, Japan). Glass transition temperatures (*T*_g_), onset of crystallization temperatures (*T*_c_) and peak crystallization temperatures (*T*_p_) were determined from the data as described by Clupper and Hench (2003).

### 2.5 Determination of bioactive glass density

Bioactive glass density was determined using the Archimedes principle. Cylindrical samples (12 mm diameter) were produced from each glass block and were suspended from a thin wire on a beaker filled with distilled water, placed on a precision weighing balance. Finally, equation 1 was used to calculate glass density:

(1) $$\rho_{s}=\rho_{fl}\cdot \frac{m_{s}}{m_{fl}}$$

where *ρ*_s_ is the density of the sample, *ρ_fl_* is the density of distilled water at room temperature, *m_s_* is the mass of the sample, and *m_fl_* is the mass of the displaced fluid.

### 2.6 Estimation of bioactive glass density using Doweidar's model

Bioactive glass density can be estimated using Doweidar's model of glass density (Doweidar, 1996, 1999) from the volume, mass and molar composition of the *Q^n^* units in the glass structure. A *Q^n^* unit is formed by one Si^4+^, *n*/2 bridging oxygen (BO), (4 – *n*) non‐bridging oxygen (NBO) and (4 – *n*) alkali metal or (4*n* – 2)/2 alkaline earth cations. The addition of a modifier oxide, *R_2_O*/*RO*, to the glass composition, where *R* is an alkali or an alkaline earth metal, creates NBOs in the SiO_2_ network, modifying the number of NBOs in a way that depends on the concentration of the modifier oxide. Doweidar hypothesized that all *Q* ^4^ units are transformed into *Q* ^3^ before *Q* ^2^ units are formed, and all *Q* ^3^ units are transformed into *Q* ^2^ before *Q* ^1^ units are formed. In bioactive glasses, the total *R_2_O/RO* concentration falls within the region 33.3 < *R_2_O/RO* < 50 mol%, resulting in a *Q^n^* unit formation model of *Q* ^3^ to *Q* ^2^. Therefore, glass density can be calculated using equation 2:

(2) $$\rho =\frac{\left(2-4x_{t}\right)\left(\sum_{i}\frac{x_{i}}{x_{t}}M_{i}^{3}\right)+\left(3x_{t}-1\right)\left(\sum_{i}\frac{x_{i}}{x_{t}}M_{i}^{2}\right)}{\left(2-4x_{t}\right)\left(\sum_{i}\frac{x_{i}}{x_{t}}V_{i}^{3}\right)+\left(3x_{t}-1\right)\left(\sum_{i}\frac{x_{i}}{x_{t}}V_{i}^{2}\right)}$$

where *i* = *a*, *b*, *c*, *x_a_*, *x_b_* and *x_c_* are the molar fractions, *x_t_* = *x_a_* + *x_b_* + *x_c_*, *M_n_^a^* , *M_n_^b^* and *M_n_^c^* are the masses, and *V_n_^a^*, *V_n_^b^* and *V_n_^c^* are the volumes of Na_2_O, SrO and CaO *Q^n^* units, respectively.

### 2.7 Calculation of bioactive glass oxygen density

Bioactive glass oxygen density may be used to evaluate the compactness of the glass network, and it can be calculated using equation 3:

(3) $$\rho_{O}=\frac{M_{O}\times \left(2x_{SiO_{2}}+5x_{P_{2}O_{5}}+x_{Na_{2}O}+x_{CaO}+x_{SrO}\right)}{\left[x_{SiO_{2}}M_{SiO_{2}}+x_{P_{2}O_{5}}M_{P_{2}O_{5}}+x_{Na_{2}O}M_{Na_{2}O}+x_{CaO}M_{CaO}+x_{SrO}M_{SrO}\right]\times \rho_{exp}^{-1}}$$

where $x_{SiO_{2}}$, $x_{P_{2}O_{5}}$, $x_{Na_{2}O}$, *x_CaO_* and *x_SrO_* are the molar fractions of SiO_2_, P_2_O_5_, Na_2_O, CaO and SrO, respectively; *M_O_* is the atomic mass of oxygen; $M_{SiO_{2}}$, $M_{P_{2}O_{5}}$, $M_{Na_{2}O}$, *M_CaO_* and *M_SrO_* are the masses of SiO_2_, P_2_O_5_, Na_2_O, CaO and SrO, respectively, and *ρ_exp_* is the density of the glass.

### 2.8 Study of bioactive glass solubility

Bioactive glass solubility was studied using a method based on ISO6872, ‘Dentistry – Ceramic Materials’. Briefly, 10 discs (12 ± 0.2 mm diameter; 1.6 ± 0.1 mm thickness) were produced from each glass block, washed in distilled water and dried at 150°C in an electric furnace for 4 h, and their mass and total surface area were determined. All samples were immersed in 100 ml 4% v/v acetic acid (Fisher Scientific, UK) solution at 80°C for 16 h. Afterwards, the samples were washed, dried and weighed to determine total loss of mass/unit surface area.

### 2.9 Energy‐dispersive X‐ray spectroscopy (EDS) analysis

Qualitative EDS analysis was used to study the elemental chemical composition of the bioactive glass discs, before and after the solubility tests were performed. All samples were sputter‐coated with carbon and analysed on a Jeol JSM6400 SEM equipped with an Oxford Instruments INCAx‐sight energy dispersive X‐ray spectrometer, operated at 20 kV. The EDS patterns generated were processed using the INCAEnergy software (Oxford Instruments, UK).

### 2.10 Isolation of mesenchymal stromal cells

Bone marrow mesenchymal stromal cells (BM‐MSCs) were isolated from 4–5 week‐old male Wistar rats, following the method described by Maniatopoulos *et al*. (1988). The femora of five individuals were dissected under aseptic conditions, cleaned of soft tissues and immersed in 10 ml Dulbecco's modified Eagle's medium (DMEM; Sigma Aldrich, UK), supplemented with 100 U/ml penicillin (Sigma Aldrich) and 1 mg/ml streptomycin (Sigma Aldrich). The ends of the femora were removed and the bone marrows were flushed into 5 ml DMEM supplemented with 10 U/ml penicillin, 0.1 mg/ml streptomycin, 20 mm l‐glutamine (Sigma Aldrich) and 10% v/v fetal calf serum (FCS; Biosera, UK). The suspended cells were seeded into 75 cm^2^ culture flasks containing 10 ml cell culture medium and incubated at 37°C and 5% CO_2_ for 24 h. Afterwards, the non‐adherent cell populations and debris were washed away with fresh cell culture medium. The cell cultures were inspected daily and the medium was changed every 48–72 h. Once the cultures achieved near‐confluence, the adherent cells were detached using a solution of 0.05% trypsin/0.02% ethylenediaminetetra‐acetic acid (Sigma Aldrich), pooled into a single population and seeded for experiments or stored for later use.

### 2.11 Cytotoxic effect of bioactive glass dissolution on BM‐MSCs

The cytotoxic effect of bioactive glass dissolution was determined using a resazurin dye‐based assay, which measures metabolic activity by monitoring a colorimetric change associated with dye reduction due to cellular respiration. The aim of this experiment was the determination of the minimum amount of bioactive glass powders inducing a significant reduction in cellular metabolic activity, which was then used in the following experiment. For this, monolayer cultures of BM‐MSCs were prepared in six‐well culture plates in triplicate, by seeding 1.5 × 10^5^ cells [passage 3, (P3)] in 2 ml/well DMEM supplemented with 10 U/ml penicillin, 0.1 mg/ml streptomycin, 20 mm l‐glutamine and 10% v/v FCS. All plates were incubated at 37°C and 5% CO_2_ for 24 h. Meanwhile, bioactive glass powders were heat‐sterilized in a dry oven at 160°C for 2 h, and samples containing 5, 10, 20, 40, 80, 160 and 320 mg were prepared in sterile Eppendorf tubes. These samples were placed in cell culture inserts (0.4 µm pore size; Greiner Bio‐One, UK) and then set on top of the monolayer cultures after the initial 24 h incubation period of BM‐MSCs was finished. A volume of 3 ml fresh culture medium was then added to each well (i.e. 2 ml on well and 1 ml on cell culture insert) and the plates were incubated at 37°C and 5% CO_2_ for 72 h. Following the removal of the medium, 2 ml solution of 10% v/v resazurin dye in fully supplemented cell culture medium were added to each well. The plates were then incubated for 100 min and two samples of cell culture medium (200 µl each) were taken from each well and transferred to 96‐well plates for spectrophotometric analysis. Fluorescence emission intensities were calculated by measuring emission at 590 nm following excitation at 560 nm, and then using equation 4:

(4) $$F_{E}=\left(F_{C}–F_{CF}\right)$$

where *F_E_* is the intensity of fluorescence emission, *F_C_* is the mean value of the fluorescence emission obtained from the wells containing cells, and *F_CF_* is the mean value of the fluorescence emission obtained from the wells containing cell‐free controls. Finally, the amount of bioactive glass powder which caused a 50% inhibition of cellular metabolic activity was determined by calculating the best‐fit curve for the data points between 0 and 80 mg for each glass composition.

### 2.12 Osteogenic effect of bioactive glass dissolution on BM‐MSCs

The osteogenic effect of bioactive glass dissolution was studied by measuring the levels of expression of genes associated with the process of osteoblastic differentiation of BM‐MSCs in standard and osteogenic cell culture media conditions. Monolayer cultures of BM‐MSCs were prepared in triplicate as previously described. Samples containing 20 mg bioactive glass powders were prepared, sterilized, placed in cell culture inserts (0.4 µm pore size, Greiner Bio‐One, UK) and then set on top of the BM‐MSCs monolayer cultures after the initial 24 h period of BM‐MSCs culture incubation had finished. A volume of 3 ml standard cell culture medium (i.e. minimum essential medium, α‐modification (α‐MEM; Sigma Aldrich, UK) supplemented with 10 U/ml penicillin, 0.1 mg/ml streptomycin, 20 mM L‐glutamine and 10% v/v FCS) or osteogenic cell culture media (i.e. same composition as the standard medium plus 50 μg/ml ascorbic acid (Sigma Aldrich, UK), 10 mM β‐glycerophosphate (Fluka Biochemika, UK) and 10^–8^ M dexamethasone (Sigma Aldrich, UK)) were added to each well (i.e. 2 ml on well and 1 ml on cell culture insert), and the plates were incubated at 37°C and 5% CO_2_ until they were terminated at days 1, 3 and 6. Total RNA content was isolated at these time points using the RNeasy Mini Kit (Qiagen, UK). RNA concentration was determined using a Nanodrop 1000 spectrophotometer (Thermo Scientific, UK) and then it was reverse‐transcribed using the High Capacity cDNA RT Kit (Applied Biosystems, UK), following the manufacturer's instructions. Reverse‐transcription quantitative polymerase chain reaction (qRT–PCR) analyses for the genes *Runx2* (Runt‐related transcription factor 2), *Alpl* (alkaline phosphatase), *Col1a1* (collagen type I *α*‐chain), *Bglap* (osteocalcin), *Bmp2* (bone morphogenetic protein 2) and *Spp1* (osteopontin) were performed on a 7900HT Fast Real‐Time PCR System (Applied Biosystems, UK), using TaqMan Gene Expression Assays (Applied Biosystems, UK). The *Gapdh* (glyceraldehyde 3‐phosphate dehydrogenase) gene was used as endogenous control in all the analyses. All the data was processed using the comparative *C* t method (i.e. *ΔΔC* t method). Fold change values were normalized to the expression levels of each gene in the control samples, which were BM‐MSCs cultured in standard cell culture medium and not exposed to bioactive glass powders.

### 2.13 Statistical analyses

Statistical analyses were performed on Microsoft Excel 2010 software, using one‐way ANOVA, followed by two‐tailed Student's *t*‐test in order to determine significance. In the study of the osteogenic effect of bioactive glasses, statistical analyses were performed using the Wilcoxon two‐group test. All results were expressed as mean ± standard deviation (SD) and considered significant at the *p* < 0.05 level.

## 3 Results

### 3.1 Particle size analysis and scanning electron microscopy imaging

**Figure 1:**
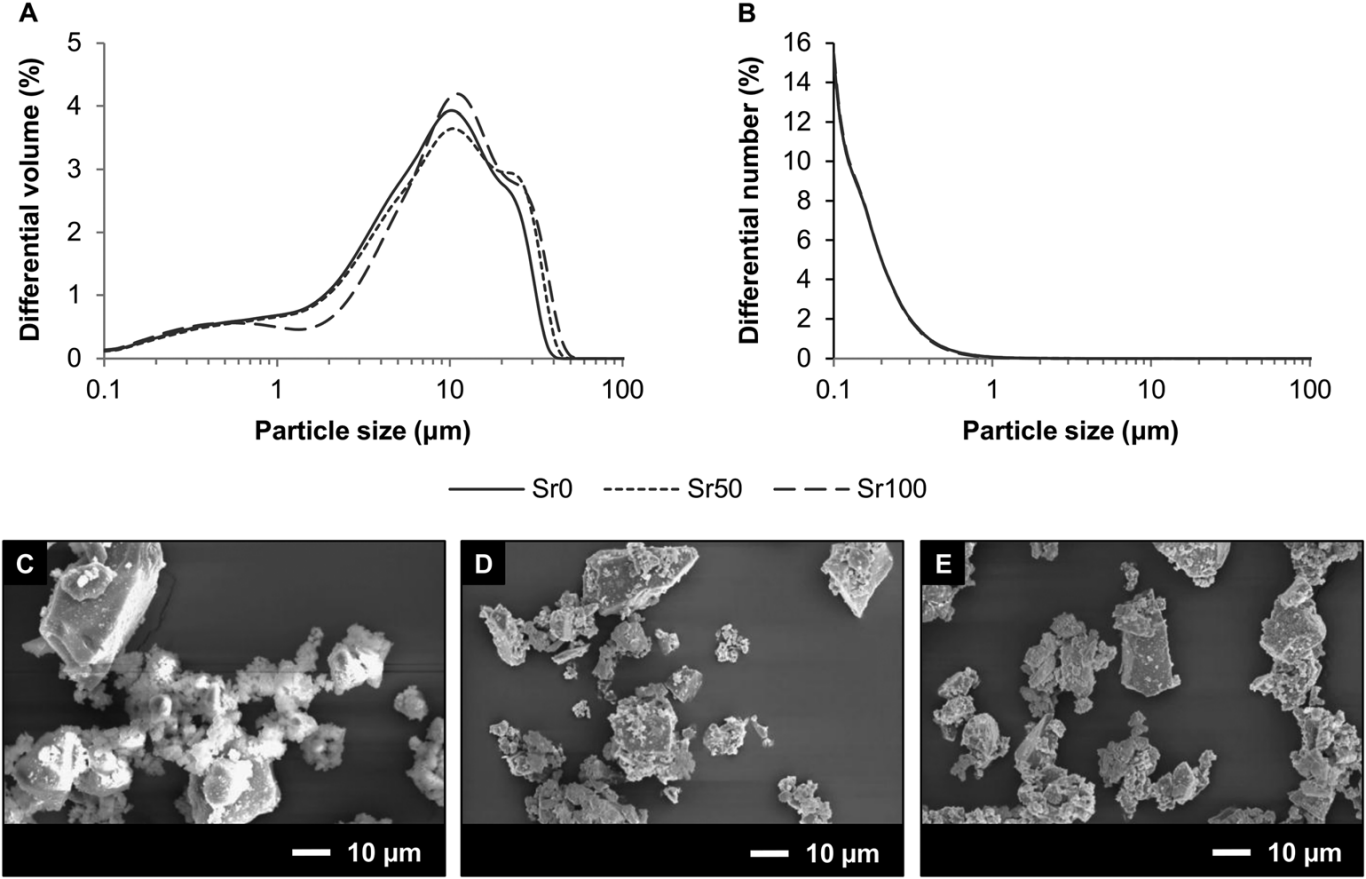
(A) Differential volume (%) distribution of particle sizes of Sr0, Sr50 and Sr100 bioactive glass powders. (B) Differential number (%) distribution of particle sizes of Sr0, Sr50 and Sr100 bioactive glass powders. Only one line can be observed in (B) due to the overlap of the data for the three samples. (C–E) SEM images of Sr0, Sr50 and Sr100 bioactive glass powders, showing great variation in particle shape and size within each sample

The differential volume distributions showed a peak centred at particle size values within the range 9–13 µm and a tail region which extended to particle sizes < 1 µm for the three bioactive glass powders (Figure 1A). The differential number distributions showed that the most abundant particles were those with sizes < 1 µm, with no observable significant differences between the three glass compositions (Figure 1B). SEM micrographs (Figure 1C–E) showed that the bioactive glass powders were composed of particles exhibiting irregular shapes and a wide range of sizes, with the smaller particles tending to form agglomerates or to attach to the surfaces of larger particles.

### 3.2 XRD and differential thermal analyses

**Figure 2:**
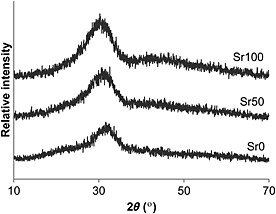
X‐ray diffraction spectra of Sr0, Sr50 and Sr100 bioactive glasses

The XRD spectra showed that the bioactive glasses were amorphous and free of any significant crystalline phases (Figure 2). The diffraction maxima in the spectra were observed to move progressively to smaller values of angle 2*θ* with the increase of strontium substitution in the glass composition. Table 2 presents the values for glass transition temperature (*T*_g_), onset of crystallization temperature (*T*_c_) and peak crystallization temperature (*T*_p_) estimated from the DTA traces for the three bioactive glass compositions. It was observed that *T*_g_ decreased as the level of strontium substitution increased from Sr0 to Sr100 compositions, while both *T*_c_ and *T*_p_ presented minimum values in the case of Sr50. Finally, the working range, calculated by subtracting *T*_g_ from *T*_c_, was observed to increase with the level of strontium substitution.

**Table 2:** Glass transition temperature (*T*_g_), onset of crystallization temperature (*T*_c_), peak crystallization temperature (*T*_p_) and working range of Sr0, Sr50 and Sr100 bioactive glasses, as determined by differential thermal analysis (DTA)

Characteristic temperature (°C)	Bioactive glasses		
	Sr0	Sr50	Sr100
Glass transition temperature (*T*_g_)	525	492	479
Onset of crystallization temperature (*T*_c_)	613	583	610
Peak crystallization temperature (*T*_p_)	682	671	691
Working range (*T*_c_–*T*_g_)	88	91	131

### 3.3 Bioactive glass density and oxygen density

**Figure 3:**
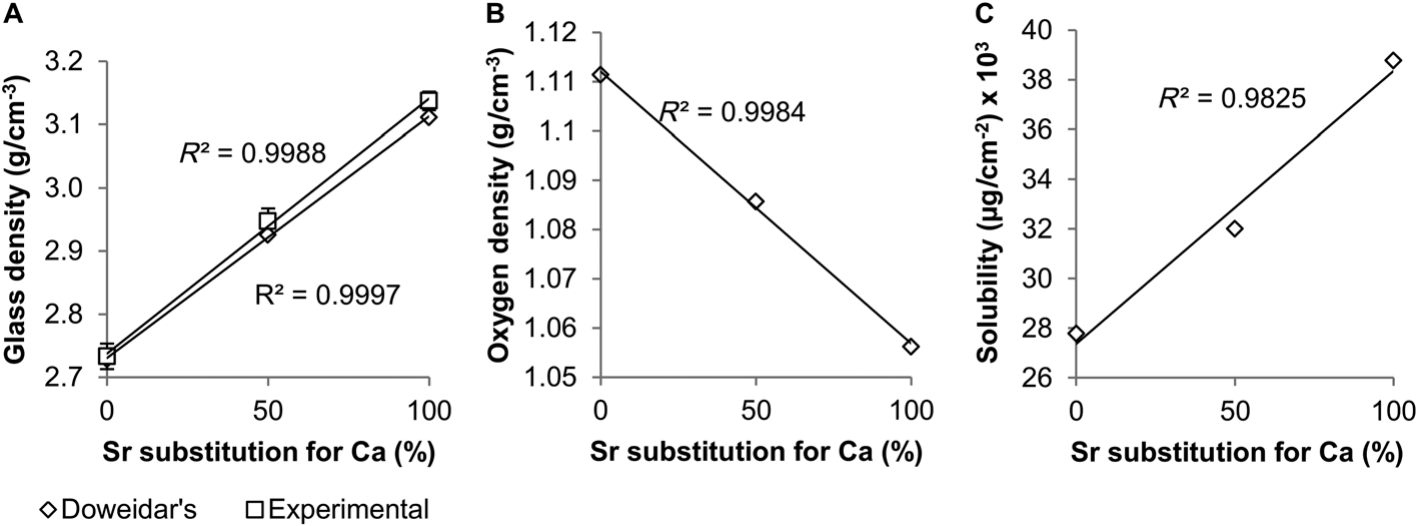
(A) Experimentally measured density, and density calculated using Doweidar's model of glass density, of Sr0, Sr50 and Sr100 bioactive glasses. (B) Oxygen density of bioactive glass plotted in relation to the level of strontium substitution in its composition. (C) Bioactive glass solubility plotted in relation to the level of strontium substitution in its composition

The experimentally measured density of the bioactive glasses was observed to increase linearly in proportion with the level of strontium substitution in the glass composition (Figure 3A) from 2.73 ± 0.02 g/cm^3^ for Sr0 to 3.14 ± 0.01 g/cm^3^ for Sr100. Glass density calculated using Doweidar's model generally showed a good agreement with the experimentally measured density. Finally, oxygen density showed a linear decrease in proportion with the strontium substitution (Figure 3B), decreasing from 1.11 g/cm^3^ for Sr0 to 1.06 g/cm^3^ for Sr100.

### 3.4 Solubility study and EDS analyses

**Figure 4:**
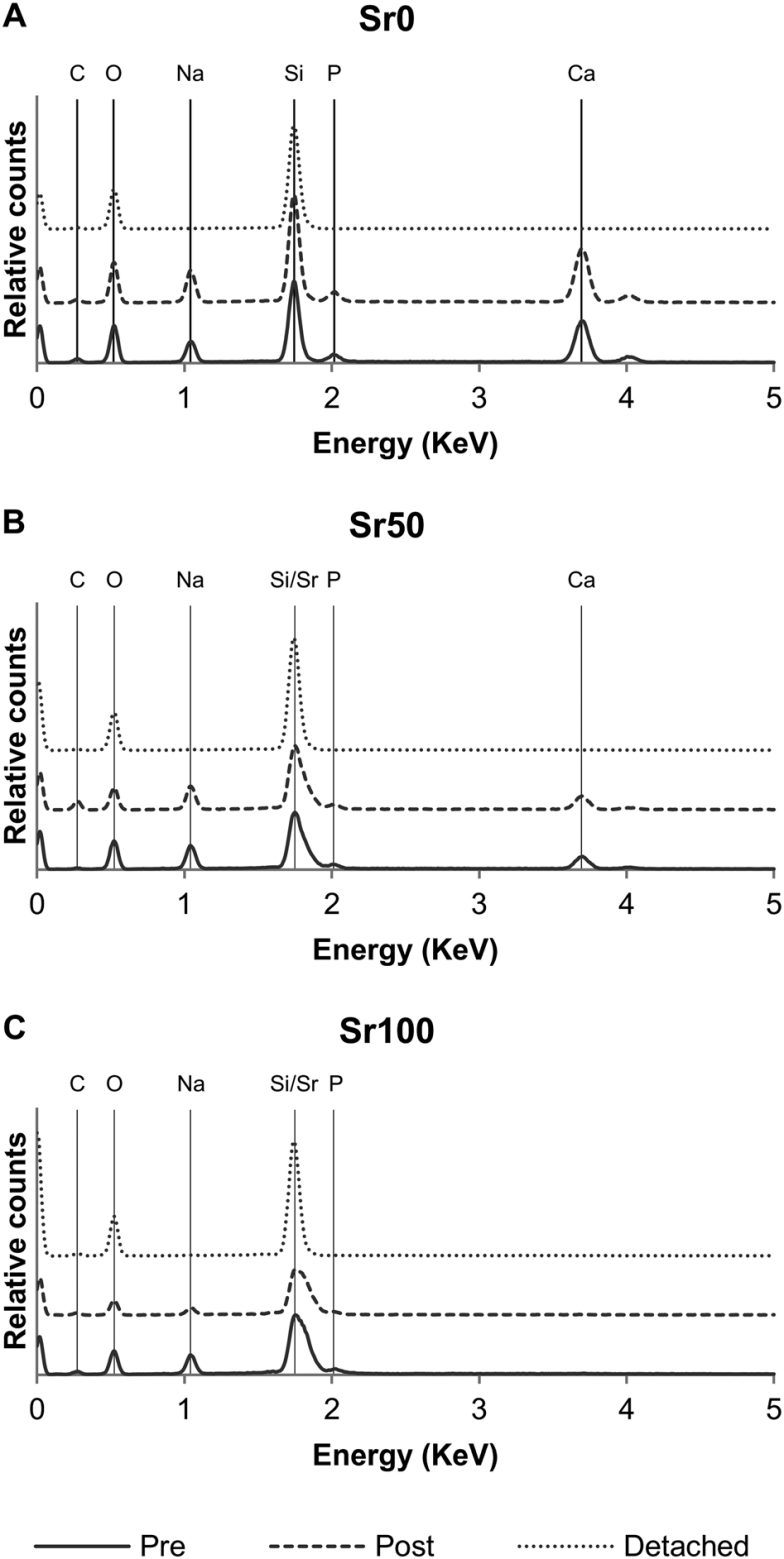
Energy‐dispersive spectra patterns for (A) Sr0, (B) Sr50 and (C) Sr100 bioactive glass samples, before and after the solubility studies were performed: Pre, bioactive glass surface before the solubility study was performed; Post, bioactive glass surface after the solubility study was performed and the modified surface layer was removed; Detached, sample of the modified and detached bioactive glass surface layer after the solubility study was performed

Bioactive glass solubility increased linearly in proportion with the level of strontium substitution in the glass composition (Figure 3C). It was observed that the appearance of the bioactive glass samples had visibly changed after being exposed to the acetic acid solution used in the solubility study, developing a surface layer which had varied in colour. Additionally, the altered surface layer was more brittle, being easily removed from the samples by mechanical action and exposing an apparently unchanged surface. EDS analyses of the samples (Figure 4) confirmed the presence of the various chemical elements forming each bioactive glass composition and it was possible to differentiate between the three of them. The altered surface layer was shown to be composed of silicon and oxygen, while the newly exposed surface exhibited an identical elemental composition to the bioactive glass samples before the study.

### 3.5 Cytotoxic effect of bioactive glass dissolution on BM‐MSCs

**Figure 5:**
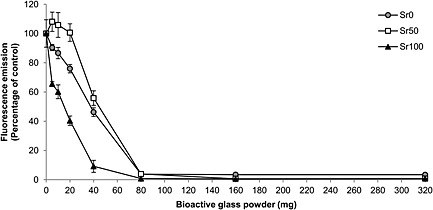
Fluorescence emission levels obtained from cell culture media used in the study of the cytotoxic effect of increasing amounts (i.e. 0–320 mg in 3 ml of medium) of Sr0, Sr50 and Sr100 bioactive glass powders. After an incubation period of 72 h in the presence of bioactive glass powders, a resazurin dye‐based assay was used to measure the metabolic activity of a monolayer culture of MSCs. Statistically significant differences with the control samples (*p* < 0.05) were observed when using amounts of glass powder ≥ 40 mg for Sr0 and Sr50, and when using amounts ≥ 20 mg for Sr100

The levels of fluorescence emission (Figure 5) showed a general decrease as the amount of bioactive glass powders used increased to 80 mg (i.e. 26.7 mg/ml). Additionally, differences between the three glass compositions were observed within that range. A significantly faster rate of decrease was observed in those samples exposed to Sr100 than in those exposed to the other two glass compositions, and the levels of fluorescence emission in samples exposed to up to 20 mg (i.e. 6.67 mg/ml) of Sr50 were greater than in the control samples (i.e. monolayer cultures of BM‐MSCs with no bioactive glass powders). Statistically significant differences (two‐tailed Student's *t*‐test, *p* < 0.05) were observed between the control samples and the samples exposed to bioactive glass powders when amounts of Sr0 and Sr50 of 40 mg (i.e. 13.33 mg/ml) and greater were used. In the case of Sr100, statistically significant differences (two‐tailed Student's *t*‐test, *p* < 0.05) were observed when amounts of 20 mg (i.e. 6.67 mg/ml) and greater were used. Finally, it was observed that Sr50 required the greatest amount of glass powder to induce a 50% inhibition, while Sr100 required the smallest. The amount of bioactive glass powder which caused a 50% inhibition in cellular metabolic activity, the equations of the best‐fit curves for the data points used, and the *R* ^2^ values for each glass composition are shown in Table 3.

**Table 3:** Amount of Sr0, Sr50 and Sr100 bioactive glass powder (in mg and mg/ml) required to induce a 50% inhibition of cellular metabolic activity *in vitro*. The equations of the best‐fit curves for the data points in the range 0–80 mg and the *R* ^2^ values are included

Bioactive glass	Amount	Equation of best‐fit curve	*R* ^2^ value
Sr0	40.18 mg	*y* = –1.198*x* + 98.134	0.996
	13.39 mg/ml		
Sr50	51.49 mg	*y* = –0.0066*x* ^2^ – 0.8154*x* + 109.48	0.961
	17.16 mg/ml		
Sr100	12.42 mg	*y* = 105.36*e* ^–0.06*x*^	0.994
	4.14 mg/ml		

### 3.6 Osteogenic effect of bioactive glass dissolution on BM‐MSCs

**Figure 6:**
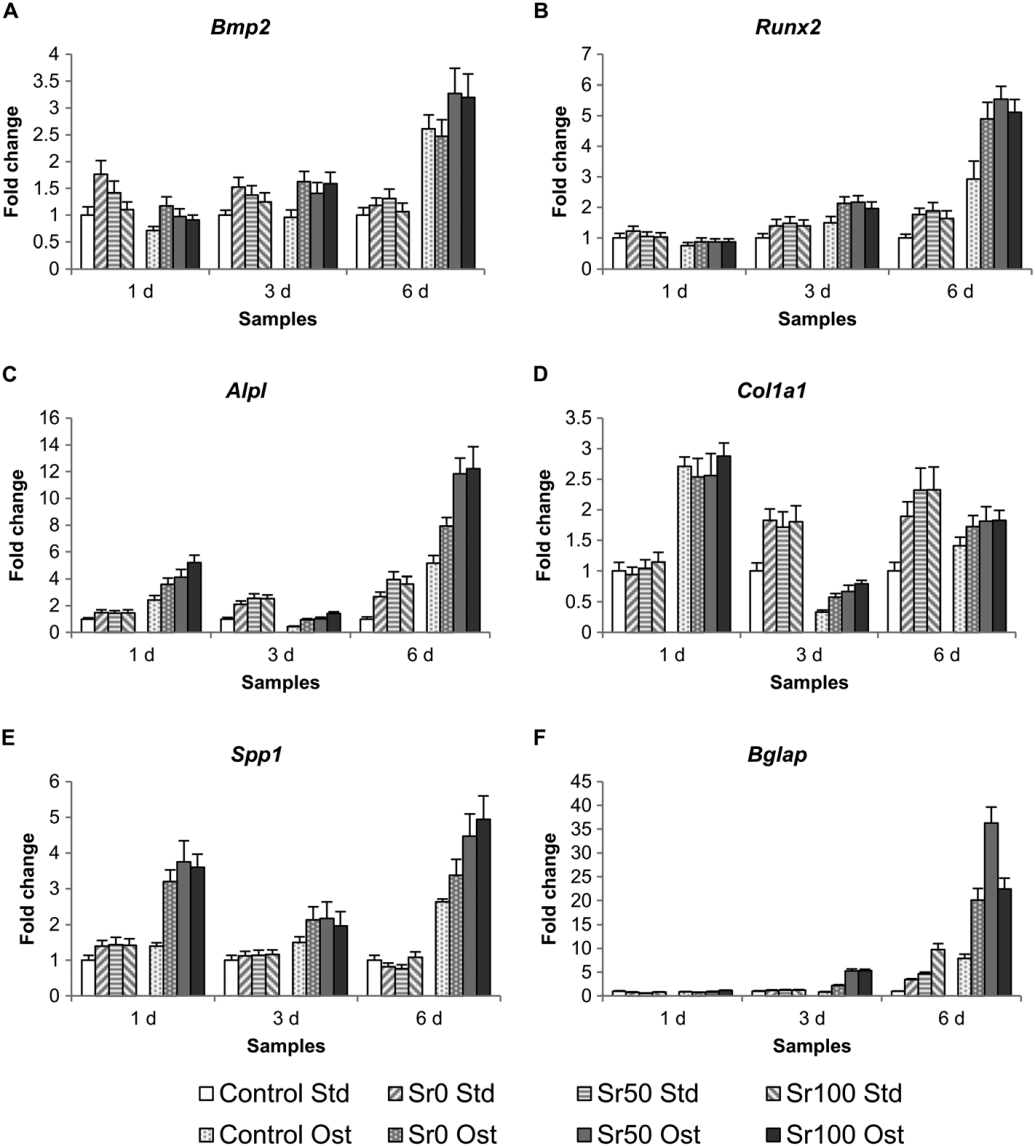
qRT–PCR analyses of the expression levels for selected genes associated with the osteoblastic differentiation process of BM‐MSCs cultured in standard cell culture medium (Std) and osteogenic cell culture medium (Ost) at 1, 3 and 6 days of exposure to 20 mg Sr0, Sr50 and Sr100 bioactive glass powders: (A) *Bmp2*; (B) *Runx2*; (C) *Alpl*; (D) *Col1a1*; (E) *Spp1*; and (F) *Bglap*. All fold changes were normalized to the values of the expression of each gene in the control samples (i.e. BM‐MSCs cultured in standard cell culture medium and not exposed to any amount of bioactive glass powder)

Figure 6 shows the levels of expression of the six genes studied on BM‐MSCs using qRT–PCR, *Runx2*, *Alpl*, *Col1a1*, *Bglap*, *Bmp2* and *Spp1*. Variations in their expression were observed regarding cell culture duration, medium conditions and bioactive glass composition. In standard cell culture medium conditions, *Runx2*, *Alpl*, *Col1a1* and *Bglap* showed a gradual increase of expression levels from day 1 to day 6, while *Bmp2* and *Spp1* showed a decrease. Statistically significant differences (Wilcoxon two‐group test, *p* < 0.05) between the samples exposed to bioactive glass powders and the controls were reported for the genes *Runx2*, *Alpl* and *Col1a1* at days 3 and 6 for the three glass compositions. In osteogenic cell culture medium conditions, *Bmp2*, *Runx2* and *Bglap* also showed a gradual increase of expression levels from day 1 to day 6, resulting in greater levels of expression than in standard cell culture medium conditions. It was also observed that the expression levels of *Alpl*, *Col1a1* and *Spp1* decreased from day 1 to day 3, later increasing at day 6. Statistically significant differences (Wilcoxon two‐group test, *p* < 0.05) between the samples exposed to bioactive glass powders and the controls were reported for all genes except *Bmp2* at days 3 and 6 for the three glass compositions. Finally, the potentially stimulatory effect of strontium was studied by comparing the levels of expression in samples exposed to Sr50 and Sr100 bioactive glass powders with those exposed to Sr0, which contained no strontium. In standard cell culture medium conditions, statistically significant differences (Wilcoxon two‐group test, *p* < 0.05) were observed for *Alpl* (days 3 and 6 for Sr50, and day 6 for Sr100), C*ol1a1* (day 6 for Sr50 and Sr100), and *Bglap* (day 6 for Sr100). Interestingly, the levels of expression of *Bmp2* at day 1 for Sr50 and Sr100 were lower than for Sr0. In osteogenic cell culture medium conditions, statistically significant differences (Wilcoxon two‐group test, *p* < 0.05) were observed for *Bmp2* (day 6 for Sr50 and Sr100), *Alpl* (day 6 for Sr50 and days 1, 3 and 6 for Sr100), *Spp1* (day 6 for Sr50 and days 3 and 6 for Sr100) and *Bglap* (day 6 for Sr50 and day 3 for Sr100).

## 4 Discussion

The characterization of the three bioactive glasses suggested that the substitution of calcium by strontium in the 45S5 composition resulted in an expansion of the glass network, most likely in order to accommodate the larger strontium cation. This is supported by the progressive movement of the XRD diffraction maxima in the spectra (Figure 2) toward smaller angle 2*θ* values, indicating greater spaces between the atoms of the glass network, according to Bragg's law; and by the decreased glass transition temperatures (*T*_g_) reported by DTA (Table 2), a likely consequence of the reduction in the thermal energy required for the transition from the glassy to the liquid state to occur due to a weakened network. Additionally, the onset of crystallization temperature (*T*_c_) and the peak crystallization temperatures (*T*_p_) were also modified with strontium substitution, leading to an increase in the working range of the glasses. Therefore, strontium‐substituted bioactive glasses may be less difficult to process at temperatures > *T*_g_ into coatings and porous scaffolds than the 45S5 composition, which is prone to crystallization during sintering, due to low silica and high calcium content (Jones, 2013; O'Donnell *et al*., 2010). Glass density increased in proportion to strontium substitution (Figure 3A), with a generally good agreement observed between the values of density measured experimentally and those calculated using Doweidar's model of glass density. Doweidar (1996, 1999) hypothesized that in alkali–silicate glasses the volumes of the *Q^n^* structural units depend only on the type of modifier ions present in the composition, but not on their concentration, making it possible to calculate the volume of the units as a function of the radius and charge of the modifier ion, and the number of non‐bridging oxygens. The agreement between both sets of density values confirmed that the volume of the *Q^n^* units with strontium was greater than the volume of the equivalent *Q^n^* units with calcium, due to the larger atomic radius of the strontium cation (Table 4). Although a greater volume may appear contrary to an increase in density, the effect of the substitution on glass density may be explained by the proportionally greater effect of the atomic mass of strontium. The expansion of the glass network due to strontium was also indicated by the linear decrease of oxygen density (Figure 3B) and the linear increase of glass solubility (Figure 3C). In both cases the likely cause is the larger strontium cation, whose presence results in a less rigidly bonded glass network and in greater rates of release of the modifier ions. This is consistent with the larger volumes of the *Q^n^* units with strontium, and is in general agreement with Fredholm *et al*. (2010). O'Donnell *et al*. (2010) argued that the increased solubility may have a potentially intensifying effect on glass reactivity and on bioactivity, but only if the substitution is done on a molar basis. If done on a weight basis, as in the work of Lao *et al*. (2008, 2009) and Gorustovich *et al*. (2010), the contents of other components in the glass (e.g. silica) will increase, resulting in a more polymerized network and in reduced solubility, degradation rates and bioactivity. However, in molar basis substitutions the network structure will not be significantly changed and bioactivity may be retained. O'Donnell *et al*. (2010) also suggested that the bioactivity of strontium‐substituted bioactive glasses may even be greater, due to the potentially stimulating effect of strontium on bone‐forming cells. The expanding effect of strontium on the glass network was also highlighted by Wu *et al*. (2011), who reported greater solubility and enlarged pore sizes in mesoporous Sr–SiO_2_ glasses as the content of strontium increased. EDS analyses (Figure 4) confirmed that dissolution began at the surface of the samples, as expected for bioactive glasses, and suggested that the modifier ions were released in dissolution, since the remaining elements on the altered surface were mainly silicon and oxygen. Particle size analyses of the bioactive glass powders (Figure 1A, B) showed no significant differences between the three compositions, due to strontium substitution. The smaller particles may aggregate together or attach to the surface of larger particles (Figure 1C–E), forming clusters that may be identified as particles of greater size during the analyses.

**Table 4:** Ionic radius and atomic mass of sodium (Na), calcium (Ca) and strontium (Sr)

Element	Ionic radius (nm)	Atomic mass (amu)
Na	0.095–0.102	22.99
Ca	0.094–0.106	40.078
Sr	0.110–0.127	87.62

Adapted from Doweidar (1999).

The study of the cytotoxic effect of bioactive glass dissolution suggested that increasing the amount of glass powders used and the level of strontium substitution in the glass composition may result in significant variations in cellular metabolic activity (Figure 5). This may be due initially to the greater solubility of the modified glasses and to the associated increase in media pH. BM‐MSCs exposed to Sr100 bioactive glass powders showed a significantly faster decrease in metabolic activity than those exposed to the other two compositions. However, the results also showed a decreased cytotoxic effect for Sr50, with cells exposed to amounts of bioactive glass powder up to 20 mg (i.e. 6.67 mg/ml) exhibiting greater values of fluorescence emission than the control samples. Additionally, the amount of bioactive glass powder required to induce a 50% inhibition of cellular metabolic activity (Table 3) was greater for Sr50 than for the other two compositions. It is possible that, in samples containing < 20 mg glass powder, Sr50 may present a balance between the cytotoxic effect of the increased solubility and the enhancing effects of strontium, as suggested by O'Donnell *et al*. (2010). They reported greater proliferation of human osteosarcoma cells exposed to the dissolution products of a glass similar to Sr50 than in exposure to unmodified glasses or to compositions similar to Sr100, implying that a particular ratio in the calcium and strontium contents in bioactive glasses may result in optimal cellular activity. Additionally, Isaac *et al*. (2011) reported increased viability of mouse osteoblastic cells after exposure to 5 wt% strontium‐substituted bioactive glass for 24 h compared to control samples (i.e. cells cultured without bioactive glass particles) and in cells exposed to other glass compositions (i.e. 0 wt% and 1 wt% substitution). However, the glasses used by Isaac *et al*. (2011) were pre‐incubated, potentially diminishing their enhancing effect before exposure to cells, due to the patial loss of their ionic content during the pre‐incubation step. It is likely that non‐pre‐incubated glasses may have a more prolonged effect, as they may take longer to release their complete ionic content. Additionally, the cells used by both Isaac *et al*. (2011) and O'Donnell *et al*. (2010) were already differentiated cells (i.e. mouse osteoblasts) and cell lines (i.e. human osteosarcoma cells). It is our hypothesis that undifferentiated primary cells such as BM‐MSCs may exhibit a different response and may generate data of more relevance to tissue engineering and regenerative medicine, especially in situations where the therapeutic intervention needs to maximize local osteostimulatory effects. For example, Wu *et al*. (2011) reported greater alkaline phosphatase activity in human MSCs exposed to mesoporous Sr–SiO2 glass containing 10 mol% strontium compared to those exposed to a glass with no strontium, suggesting that the presence of strontium may compensate for the negative effect of high Si^2+^ concentration. Zhu *et al*. (2013) exposed canine MSCs to a borate bioactive glass with a 6 mol% strontium substitution and reported greater proliferation than in cells exposed to glasses with greater and lower strontium content. These reports may also support the hypothesis that appropriate ratios between strontium and other components in the glass may maximize cellular response, similar to what was suggested by O'Donnell *et al*. (2010).

qRT–PCR analyses (Figure 6) suggested that the dissolution of the bioactive glass powders stimulated the upregulation of six genes associated with the process of osteogenic differentiation: *Runx2* (Runt‐related transcription factor 2), *Alpl* (alkaline phosphatase), *Col1a1* (collagen type I α‐chain), *Bglap* (osteocalcin), *Bmp2* (bone morphogenetic protein 2) and *Spp1* (osteopontin). Alkaline phosphatase activity has usually been considered to be one of the early markers of osteogenesis. *Runx2* has been identified as the major transcription factor controlling osteoblast commitment and differentiation, being expressed by MSCs at the onset of skeletal development and by osteoblasts during their differentiation. It regulates the expression of various other genes, including *Col1a1*, *Spp1* and *Bglap*. Finally, *Bmp2* promotes *Runx2* expression in mesenchymal osteoprogenitors and osteoblastic cells (Marie, 2008; Safadi *et al*., 2009). The expression of *Bmp2*, the first gene in the sequence of events, was observed to increase in the presence of bioactive glasses at day 1 in standard cell culture medium, although it then decreased gradually up to day 6. This suggested that *Bmp2* may have been stimulated by the presence of bioactive glasses even in the absence of osteogenic factors, potentially responding to the dose of the various dissolved glass components. The decrease of *Bmp2* by day 6 may be explained by the reduced concentrations of those same components as the glasses were depleted after several changes of medium. In osteogenic cell culture medium the expression of *Bmp2* showed a different pattern, increasing gradually from day 1 and peaking at day 6, suggesting that both the osteogenic factors and dissolution products may have acted synergistically in order to stimulate a greater upregulation. The expression of *Runx2* increased between days 1 and 6 in both cell culture medium conditions, an expected response if the process of osteoblast differentiation had already started. *Alpl*, *Col1a1* and *Bglap* exhibited a gradual increase in the levels of expression between days 1 and 6 in standard cell culture medium conditions. However, in osteogenic cell culture medium conditions the expression of *Alpl* and *Col1a1* peaked initially at day 1 and then decreased at day 3, finally increasing again at day 6. This suggested that the combined effect of the osteogenic factors and the initial release of the dissolution products may have an enhancing effect on the expression of these two genes. *Spp1* exhibited a similar pattern of expression to *Bmp2* in standard cell culture medium conditions, and to *Alpl* and *Col1a1* in osteogenic cell culture conditions, suggesting that *Spp1* may be significantly more affected by osteogenic factors than by bioactive glasses. However, it may also be that the combined effect of both stimuli may have an enhanced effect on its expression. In conclusion, the results suggested that the presence of bioactive glasses in cell culture medium had a stimulatory effect on the expression of osteogenic genes in the absence and presence of osteogenic factors. It was also suggested that strontium‐substituted bioactive glasses may not always have a clear enhancing effect on the expression of osteogenic genes over unmodified compositions. The clearest stimulatory effect was observed on *Alpl*, *Col1a1* and *Bglap* in standard cell culture medium conditions, and on *Bmp2*, *Alpl*, *Spp1* and *Bglap* in osteogenic cell culture medium conditions. *Alpl* and *Bglap* were the only genes simultaneously upregulated in both experimental conditions in the presence of strontium, and were the same differentiation markers reported by Xynos *et al*. (2000a) to be enhanced by bioactive glass dissolution products, suggesting the promotion of a mature osteoblast phenotype. More recently, Strobel *et al*. (2013) reported the increased expression of *Bglap*, *Col1a1* and *Runx2* in human MSCs after 14 days of exposure to cell culture medium containing nanoparticles of strontium‐substituted bioactive glass fabricated by flame spray synthesis. However, the bioactive glass content was regularly renewed throughout the experiment, as opposed to what was done in this study. Therefore, the renewal of strontium in the medium may further enhance genetic expression, as it is never completely depleted, a situation which would not occur in the case of implanted bioactive glass. While our data and other published studies indicated that strontium‐substituted glasses influence cell behaviour, it is expected that the substitution by strontium will affect the relative concentrations of other ionic species, and this too may affect biocompatibility.

Regarding the molecular mechanisms through which the enhancing effect of strontium may occur, it is known that strontium is able to promote the osteogenic differentiation of human MSCs via different molecular routes, including the Ras–MAPK (Barradas *et al*., 2012; Peng *et al*., 2009) and Wnt–Catenin (Yang *et al*., 2011) signalling pathways. According to Peng *et al*. (2009), strontium was able to induce the upregulation of Runx2 transcriptional activity and phosphorylation in human bone MSCs through the Ras–MAPK route, which in turn resulted in the upregulation of genes such as *BGLAP* and *COL2A*, and increased osteoblastic differentiation. Barradas *et al*. (2012) showed that MAPK signalling is essential for the expression of BMP‐2, with strontium acting via ERK 1/2‐ and p38‐dependent mechanisms (Peng *et al*., 2009). Yang *et al*. (2011) showed that strontium was able to induce the *in vitro* upregulation of *β*‐catenin in human umbilical MSCs, thus mediating the activation of transcription factor Runx2 and the osteogenic differentiation of the cells. Additionally, their data showed the *in vivo* upregulation of *β*‐catenin expression when using collagen/strontium‐substituted hydroxyapatite scaffolds, resulting in the enhancement of signal transduction in order to activate the expression of osteogenic transcription factors. In both studies, Peng *et al*. (2009) and Yang *et al*. (2011) showed that strontium led to increased levels of alkaline phosphatase activity. The calcium‐sensing receptor (CaSR) has also been proposed as an important participant in the enhancing effect of strontium, mainly due to the capacity of this receptor to sense other divalent cations apart from Ca^2+^ (Saidak and Marie, 2012). Yang *et al*. (2011) suggested that CaSR in MSCs may be involved in the stimulation of Wnt secretion and the upregulation of *β*‐catenin. However, studies by Barradas *et al*. (2012), targeting CaSR using various agonists including strontium, suggested that this receptor may not be involved in the expression of BMP‐2. However, there may be other G protein‐coupled receptors similar to CaSR linked to BMP‐2 and activated by both calcium and strontium cations.

## 5 Conclusions

The substitution of calcium with strontium in the composition of 45S5 bioactive glass resulted in significant modifications of various physical properties of the glasses, including density and solubility. These changes may partly account for altered *in vitro* biocompatibility, with the most soluble bioactive glass (Sr100) causing the greatest inhibition of cellular metabolic activity. However, there is evidence that strontium can play a stimulatory role, as observed in the case of BM‐MSCs exposed to amounts of Sr50 bioactive glass powders up to 20 mg, which showed greater levels of metabolic activity compared with the controls. Additionally, the presence of bioactive glass dissolution products in the cell culture environment was associated with the promotion of osteoblastic differentiation of BM‐MSCs, with evidence that strontium‐substituted bioactive glasses were able to further upregulate the expression of *Alpl* and *Bglap* genes in both standard and osteogenic cell culture media conditions. These results are very important, in that they both confirm the potentially superior regenerative properties of strontium‐substituted bioactive glasses, and support the concept that bioactive glasses may exhibit cell‐selective properties *in vitro* leading to differentiation into an osteogenic lineage. It was therefore concluded that strontium‐substituted bioactive glasses are capable of more potent bone tissue regeneration via selective cell stimulation, and therefore have significant potential for the development of improved bone graft substitutes.

## Conflict of interest

The authors have declared that there is no conflict of interest.
